# Mutations in *SOX17* are Associated with Congenital Anomalies of the Kidney and the Urinary Tract

**DOI:** 10.1002/humu.21378

**Published:** 2010-10-19

**Authors:** Stefania Gimelli, Gianluca Caridi, Silvana Beri, Kyle McCracken, Renata Bocciardi, Paola Zordan, Monica Dagnino, Patrizia Fiorio, Luisa Murer, Elisa Benetti, Orsetta Zuffardi, Roberto Giorda, James M Wells, Giorgio Gimelli, Gian Marco Ghiggeri

**Affiliations:** 1 Biologia Generale e Genetica Medica, Università di PaviaPavia, Italy; 2 Service of Genetic Medicine, University Hospitals of GenevaGeneva, Switzerland; 3 Laboratorio di Fisiopatologia dell'Uremia, Istituto G. GasliniGenova, Italy; 4 IRCCS E. Medea, 23842 Bosisio Parini (LC)Italy; 5 Division of Developmental Biology, Cincinnati Children's Hospital Medical CenterCincinnati, Ohio; 6 Laboratorio di Genetica Molecolare, Istituto G. GasliniGenova, Italy; 7 Division of Regenerative Medicine, Stem Cells and Gene Therapy, S. Raffaele Scientific InstituteMilan, Italy; 8 Laboratorio di Citogenetica, Istituto G. GasliniGenova, Italy; 9 Unità di Nefrologia, Dialisi e Trapianto, Dipartimento di Pediatria, Azienda Ospedaliera, Università di PadovaPadova, Italy; 10 IRCCS Fondazione C. MondinoPavia, Italy; 11 Divisione di Nefrologia, Istituto G. GasliniGenova, Italy

**Keywords:** congenital anomalies of the kidney, CAKUT, *SOX17*, Wnt

## Abstract

Congenital anomalies of the kidney and the urinary tract (CAKUT) represent a major source of morbidity and mortality in children. Several factors (PAX, SOX,WNT, RET, GDFN, and others) play critical roles during the differentiation process that leads to the formation of nephron epithelia. We have identified mutations in *SOX17,* an HMG-box transcription factor and Wnt signaling antagonist, in eight patients with CAKUT (seven vesico-ureteric reflux, one pelvic obstruction). One mutation, c.775T>A (p.Y259N), recurred in six patients. Four cases derived from two small families; renal scars with urinary infection represented the main symptom at presentation in all but two patients. Transfection studies indicated a 5–10-fold increase in the levels of the mutant protein relative to wild-type SOX17 in transfected kidney cells. Moreover we observed a corresponding increase in the ability of SOX17 p.Y259N to inhibit Wnt/β-catenin transcriptional activity, which is known to regulate multiple stages of kidney and urinary tract development. In conclusion, *SOX17* p.Y259N mutation is recurrent in patients with CAKUT. Our data shows that this mutation correlates with an inappropriate accumulation of SOX17-p.Y259N protein and inhibition of the β-catenin/Wnt signaling pathway. These data indicate a role of SOX17 in human kidney and urinary tract development and implicate the SOX17–p.Y259N mutation as a causative factor in CAKUT.

Hum Mutat 31:1352–1359, 2010. © 2010 Wiley-Liss, Inc.

## Introduction

Mammalian kidneys derive from two tissue compartments of the embryonic mesoderm, that is, the ureteric bud (UB), derived from the Wolffian duct, and the metanephric mesenchyme (MM), whose interaction induces the metanephric mesenchyme to trans-differentiate into nephron epithelia [Vainio and Lin, 2002; Woolf et al., 2004]. Failure of this mechanism, such as in ectopic or supernumerary ureters [Kume et al., 2000; Miyazaki et al., 2000; Nishimura et al., 1999], is considered an underlying cause of a wide variety of renal malformation [Mackie and Stephens, 1975; Pope et al., 1999]. Gene targeting experiments in mice have led to the characterization of specific regulators of both the conversion of epithelial into mesenchymal cells and the branching of the ureteric bud. The Wingless-related signaling pathway (Wnt), for example, is critical at several stages of the process including initiation of metanephric development [Carroll et al., 2005], branching [Majumdar et al., 2003], and development of the nephron [Stark et al., 1994]. Several Wnt ligands and receptors are expressed during kidney development and activation of the Wnt pathway results in the translocation of β-catenin into the nucleus [Karihaloo et al., 2005]. β-Catenin is a key transcriptional effector of the Wnt pathway, and it was recently demonstrated that loss of β-catenin/Wnt signaling in the developing Wolffian duct causes defects including ectopic ureters and renal aplasia [Marose et al., 2008]. Tight control of the Wnt pathway is critical for normal development, and several Wnt antagonists are expressed during kidney and urinary tract development. One example is the HMG-box transcription factor SOX17, which is known to inhibit canonical Wnt signaling by forming a complex with β-catenin and TCF/LEF family members and targeting them for degradation in a GSK3β-independent manner [Sinner et al., 2007]. Analysis of the Genito-Urinary Development database (http://www.gudmap.org) [McMahon et al., 2008] shows in situ hybridization and microarray expression data demonstrating that *Sox17* is expressed at several key stages during kidney and urinary development. Specifically, *Sox17* is expressed in the ureteric bud and metanephric mesenchyme of the developing kidney and urinary tract between Theiler Stage 19–23 (E11.5–15.5 days after fertilization).

Here we observe a young girl with congenital defects of the urinary tract, chronic constipation, and mild mental retardation, who carried a de novo pseudodicentric duplicated chromosome 8 of maternal origin. This gave us the opportunity to study the genes contained within the duplication, which include SOX17. We have identified a p.Y259N mutation in *SOX17* in this girl, and we subsequently identified the same mutation in five additional patients with congenital anomalies of the kidney and the urinary tract (CAKUT). Furthermore, two patients presenting with CAKUT carried other *SOX17* mutations. Functional studies of SOX17–p.Y259N demonstrated that this protein abnormally accumulates in cultured kidney cells, resulting in inhibition of the canonical Wnt signaling pathway. Given the vital role of this pathway in multiple stages of kidney development, we propose a model by which elevated levels of mutant *SOX17–*p.Y259N protein inhibit Wnt signaling resulting in abnormal kidney and urinary tract development.

## Materials and Methods

### Patients

**Figure 1:**
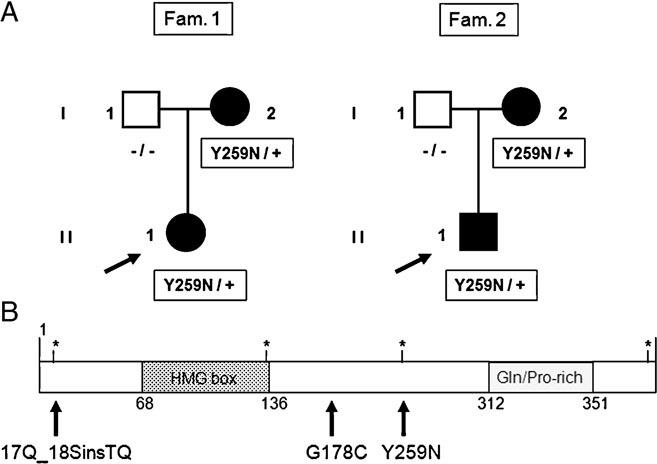
**A:** Pedigrees of affected families. Family 1: I.1 healthy father; I.2 mother with recurrent urinary infections, duplex ureter with dilatation, chronic constipation; II.1 46,XX,dirdup(8q)(q11.1q12.1)dic, duplex ureter dx, reflux dx (III degree), hydronephrosis sn, reflux (IV degree), chronic constipation, thinning of the posterior portion of the body of the corpus callosum. Family 2: I.1 Healthy father; I.2 vesicoureteral reflux; II.1 hydronephrosis sn, stenosis of the pyeloureteral joint sn. **B:** Localization of mutations along the *SOX17* gene coding sequence. The location of the HGM box and the G/P-rich region are indicated. The positions of tyrosine residues (*) predicted to undergo phosphorylation are also indicated. One of these residues is the mutated Y259.

Fifty-eight familial cases with vesico ureteral reflux (VUR) belonging to 10 small families were analyzed. In two of the families the same p.Y259N mutation in *SOX17* was found (see the Results section). Pedigrees of the two families are shown in Figure 1A.

### Family 1

Patient (II,1) was born from non consanguineous parents. The pregnancy was achieved after in vitro fertilization (FIVET). A Cesarean section at the 34th week of gestation was performed because of polydramnios and a prenatal ultrasonography showing severe bilateral hydronephrosis in the fetus. At birth, she was put on intensive care because of her critical health conditions. In the first 3 years of her life, she had presented mild axial hypotonia and psychomotor development with a deficit in expressive language. She had chronic constipation. Echocardiography showed a normal heart with large PDA and a bidirectional shunt. Encephalic RM demonstrated abnormal morphology of the corpus callosum, characterized by evident thinning of the posterior portion of the body and of the isthmus. Her right kidney showed a duplicated pyeloureteral collecting system of about 7 cm in diameter and bilateral VUR (III). Ureteroplasty was performed to correct the patient's urinary defects. The patient's mother (I,2) showed VUR associated to megaureter with recurrent urinary infections, chronic constipation, and coloboma of the iris in her left eye. Her father was healthy.

### Family 2

Patient (II,1) is a male child with left hydronephrosis due to stenosis of the pyeloureteral joint. His mother (I,2) has vesicoureteral reflux, whereas his father is healthy.

### Sporadic Cases

Additionally, 178 patients with proven VUR and a history of urinary tract infection were enrolled in the study. Control DNAs were obtained from 88 normal subjects, 82 cord blood samples from normal donors, and 135 patients with nephrotic syndrome. Although the former two groups were not controlled in respect to the presence of any defect of the urinary tract, all patients with nephrotic syndrome had an echosonography showing normality. The demographic and clinical data relative to all the above cohorts are given in Table 1.

**Table 1:** *SOX17* Mutation Analysis of Subjects with Vesicoureteral Reflux (VUR), DMSA-Positive (DMSA+UTI), and Renal Scars Associated with Recurrent Urinary Tract Infections

	Number (*n*)	Sex (M/F)	SOX17 p.Y259N *n*	SOX17 p.G178C *n*	SOX17 p.Q17_S18insTQ *n*	Diagnosis (grade, position)	Tc99m DMSA
CAKUT							
	58 (10 fam.)	26/32	4				
*Familial cases*	Fam.1, II.1	F	het			VUR (III grade, R&L) double pelvis	Renal scars
	Fam.1, I.2	F	het			VUR (IV grade, R)	Normal
	Fam.2, II.1	M	het			VUR (III grade, L)	Normal
	Fam. 2, I.2	F	het			VUR (III grade, L)	Renal scars
*Sporadic cases*	178	57/121	2	1	1		
	Pt 180	F	het			ureter dilatation L	Renal scars
	Pt 240	F	het			VUR (III grade, L)	Renal scars
	Pt 253	M		het		VUR (I grade, R&L)	Renal scars
	pt 36	M			het	VUR (III grade, R&L)	Renal scars
Controls (CT)	88	40/48	1				
	ct 1	M	het			n.a	n.a
Cord blood	82	40/42	0				
Nephrotic syndrome	135	60/75	0				

Three groups of unselected control subjects were also analyzed. One of this included children with nephrotic syndrome who were excluded for VUR. VUR=vesicoureteral reflux; UTI, urinary tract infections; n, number; M, male; F, female; het, heterozygote; L, left; R, right; Tc99m DMSA, Technetium99-dimercaptosuccinic acid; n.a, not available.

All patients with VUR had been studied with echosonography, voiding cystography and Tc99 mDMSA scan in order to verify the presence of malformations of the kidney and urinary tract and/or renal scars. Static renal scintigraphy was recorded 3 to 4 hr after injection of a weight-scaled dose of technetium-99 m DMSA to obtain views in the posterior and both posterior oblique projections for 300 kilocounts or more. Focal or diffuse areas of decreased uptake in the first scan, without evidence of cortical loss, indicated acute pyelonephritis. Renal scarring was defined as decreased uptake with distortion of the contours or as cortical thinning with loss of parenchymal volume. Two nuclear physicians, blind to the test results, interpreted the scans independently and resolved discrepancies by discussion.

### Cytogenetics, Immunofluorescence, and Array-CGH Investigations

Chromosome preparations were made from peripheral blood, skin, and ureter biopsies from the proposita using standard techniques. To define the extension of the duplicated segment an array-CGH experiment was performed using the Human Genome CGH Microarray Kits 44B (Agilent Technologies, Palo Alto, CA) covering the whole genome with a resolution of ∼100 kb. Briefly, 1 µg of patient and sex-matched pooled reference DNAs were processed according to the manufacturer's protocol. Fluorescence was scanned in a dual-laser scanner and the images were extracted and analyzed with Agilent Feature Extraction software (v9.5.3.1) and CGH Analytics software (v3.5.14), respectively. Changes in test DNA copy number at a specific locus are observed as the deviation of the log ratio value from a modal value of 0.

To confirm the breakpoints, structure, and orientation of the duplicated region we used fluorescent in situ hybridization (FISH) with BAC clones spanning the chromosomal 8q11.1q12.1 regions selected according to the University of California Santa Cruz (UCSC) Human Genome Assembly (March 2006 assembly). To investigate the activation status of the two centromeres present on the duplicated chromosome 8, immunofluorescence analysis was performed by CENP-C antibody, as described [Gimelli et al., 2000].

### SOX17 Mutation Analysis

DNA was extracted from lymphoblasts or whole blood sample with the High Pure PCR Template Preparation Kit (Roche Diagnostics, Italy). We also obtained genomic DNA from cultured skin biopsy fibroblasts and from the proband's ureteric tissue. Amplification and sequencing of the *SOX17* (NM_022454.2) gene and its promoter region were performed by PCR amplification using the following primer pairs:

*SOX17* Ex1a forward: 5′-ggccacatctgtgcagaaaa-3′
*SOX17* Ex1a reverse: 5′-CTCTGGGTCTGGCTCTGGT-3′
*SOX17* Ex1b forward: 5′-GCATCTCAGTGCCTCACTCC-3′
*SOX17* Ex1b reverse: 5′-cgtcaggctcgcaaagaa-3′
*SOX17* Ex2a forward: 5′-tgcgcaattcaaagtctgag-3′
*SOX17* Ex2a reverse: 5′-CGCCGTAGTACACGTGAAGG-3′
*SOX17* Ex2b forward: 5′-CCGGCACCTACAGCTACG-3′
*SOX17* Ex2b reverse: 5′-cacccttttcgaggatgaga-3′

All amplification reactions were performed with standard PCR conditions in a GeneAmp 9700 PCR System (Applied Biosystems, Foster City, CA). Unincorporated dNTPs and primers were removed from 5 µl of PCR product by digestion with 2 µl of ExoSAP-IT (USB, Europe GmbH). Automated sequence analysis was performed by dye-terminator reactions on an ABI3130xl (Applied Biosystems) and electropherogram analysis by Sequencer Software 4.6 (GeneCodes Corporation, Ann Arbor, MI).

### Segregation Analysis and Somatic Cell Hybrids

Parental origin of the duplication was studied by generating somatic cell hybrid clones to isolate the duplicated chromosome 8 from its normal homolog [Giorda et al., 2004]. Genomic DNA was extracted from the proposita's EBV cell line and her parents' lymphocytes using standard protocols; genomic DNA from hybrid clones was extracted using DNAzol (MRC Inc., Cincinnati, OH). Genotyping of polymorphic loci was performed by amplification with primers labeled with fluorescent probes (ABI 5-Fam, Hex and Tet) followed by analysis on a ABI 310 Genetic Analyzer (Applied Biosystems). Amplifications were performed with Taq Gold (Applied Biosystems) using standard protocols. Sequencing reactions were performed with a Big Dye Terminator Cycle Sequencing kit (Applied Biosystems) and run on an ABI Prism 3100 AV Genetic Analyzer.

### Western Blot Analysis and Luciferase Assays

HEK293T cells were obtained from ATCC and cultured in Dulbecco's modified Eagle's medium (DMEM) (Gibco, Grand Island, NY) supplemented with 10% fetal bovine serum (FBS) (Hyclone, Logan, UT). Cells were plated into 24-well plates 24 hr prior to transfection. For Western blotting, cells were transfected with the Sox17 plasmids (100–300 ng), and a GFP plasmid (100 ng) as a transfection control, using Lipofectamine 2000 (Invitrogen, Carlabad, CA) according to the manufacturer's protocol. Total amount of DNA was kept constant by adding empty vector DNA. Total cell lysates were prepared 48 hr posttransfection using RIPA buffer and analyzed by Western immunoblotting. Antibodies used were goat anti-Sox17 (1:5000, R&D Systems, Indianapolis, IN) and mouse antitubulin (1:5,000; Sigma, St. Louis, MO); the secondary antibodies were rabbit antigoat HRP (1:10,000; Vector Laboratories, Burlingame, CA), and goat antimouse HRP (1:10,000, Jackson ImmunoResearch, West Grove, PA). Each experiment was repeated at least three times and a representative sample is shown. For luciferase assays, cells were transfected with Sox17 plasmids (3–50 ng), either the TOPflash Wnt reporter plasmid or a SOX17 luciferase reporter plasmid (50 ng) [Sinner et al., 2007], a renilla plasmid (50 ng) as a transfection control, and a constitutively active *S37A* β*-catenin* plasmid (50 ng) for the TOPflash assay. The Sox17 reporter plasmid contained eight copies of a Sox17 binding site and the TOPflash reporter contains TCF/LEF binding sites and were previously described [Sinner et al., 2007; Zorn et al., 1999]. Total amount of DNA was kept constant by adding empty vector DNA, and cell lysates were prepared for luciferase assay as previously described [Sinner et al., 2007].

### Quantitative RT-PCR Analysis

For quantitative RT-PCR, total RNA was extracted from cells using a NucleoSpin RNA II Kit (Macherey and Nagel, Germany) and treated with RNase-free DNase (Roche, Indianapolis, IN). cDNA was synthesized with random hexamer primers. Real-time RT-PCR was performed using an Opticon machine (MJ Research-Bio Rad, Hercules, CA). The primers used in this study were: *hSox17 f*orward 5′ GAC GAC CAG AGC CAG ACC 3′ and *hSox17* reverse 5′ CGC CTC GCC CTT CAC C 3′; *h*β*-tubulin* forward 5′ GAT ACC TCA CCG TGG CTG CT 3′ and *h*β*-tubulin* reverse 5′ AGA GGA AAG GGG CAG TTG AGT 3′. SYBR green dye (Qiagen, Chatsworth, CA) was included in the PCR mix. The amount of product in each sample was estimated at the log-linear amplification phase, and these values were normalized to the expression level of tubulin and the data presented as a ratio of tubulin expression.

## Results

**Figure 2:**
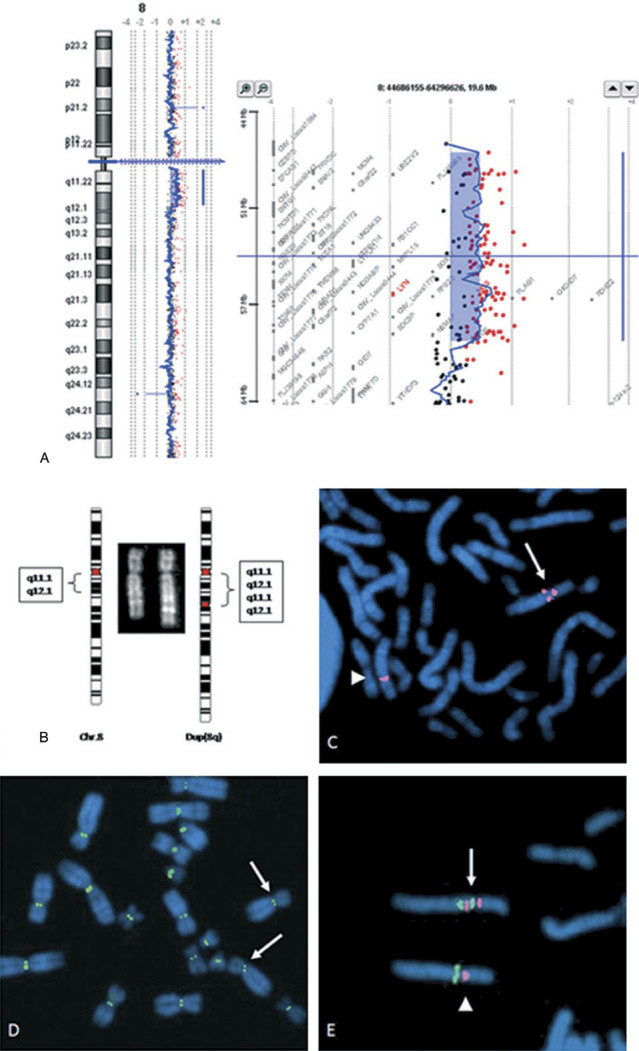
**A:** Array-CGH, cytogenetics, and FISH results. Ratio plot of high-resolution oligonucleotide array mapping of duplication 8q11.1–q12.1 (chr8:47655000-60101000) from the proband of Family 1. This technique evidenced a duplication of 13,1 Mb spanning from probe A_14_P107002 at 47.655 Mb to probe A_14_P128857 at 60.101 Mb. Because the probes contained in the Agilent 44B microarray slide do not cover the region “Gap_225” (3 Mb) from 8p11.1 to 8q11.1, between the centromere and probe A_14_P107002, we completed this interval by FISH with BAC clones. **B:** Cutout and ideogram of the normal chromosome 8 (left) and the duplicated (right) chromosome 8. Parental karyotypes were normal, suggesting that the abnormality was de novo. **C:** FISH with CEP8 (Vysis) showing the normal (arrowhead) and the dicentric duplicated chromosome 8 (arrow). In fact, the CEP 8 probe evidenced the presence of a second centromere or, most likely, a partial centromere because it appeared of reduced dimensions. **D:** After immunofluorescence with CENP-C, signals (green) are localized at the centromere of the normal chromosome 8 (arrowhead) but not at the second inactive centromere of the duplicated chromosome 8 (arrow). **E:** Dual-color FISH with BAC probes RP11-53M11, mapping to 8q11.23, and containing the gene *SOX17* (green signal), and CEP8 (Vysis), specific for alphoid DNA of chromosome 8 centromere (red signals), showing a direct duplication of the segment 8q11.1–q12.1 (arrow); arrowhead indicates normal chromosome 8.

Our study began with the observation of a young girl with congenital defects of the urinary tract, chronic constipation, and mild mental retardation, who carried a de novo pseudodicentric duplicated chromosome 8 (Family 1; Fig. 1A). The de novo duplication dup(8)(q11.1q12.1) of 13.1 Mb on chromosome 8 was demonstrated by CGH-array (Agilent 44B) and confirmed by FISH analysis using BAC clones known to map to the duplicated region (Fig. 2). A direct duplication was demonstrated by dual-color FISH with BAC RP11-53M11 and CEP8. A pseudodicentric chromosome 8 with a inactive centromere was evidenced by FISH with CEP8 and by immunostaining with CENP-C antibody (Fig. 2D). These results allow us to define the karyotype of our proposita as: 46,XX,psudicdup(8)(q11.1q12.1) (chr8:46,958,053..60,381,578).

Among the 38 genes located in the duplicated region of chromosome 8, *SOX17* seemed a good candidate to study because of its expression throughout development of the urogenital tract in the mouse and its role in regulating β-catenin signaling. We identified a point mutation at nucleotide 775 with respect to the translation start site (c.775T>A) in the *SOX17* gene. This mutation results in a protein change from tyrosine at position 259 to an asparagine residue (p.Y259N). The mutation is localized between the HMG-box and the glycine–proline-rich segment at the C-terminal of the protein (Fig. 1B). The same mutation was found in another family with VUR (out of 10 families for an overall of 58 affected) and in several sporadic subjects presenting VUR and renal scars.

Segregation analysis of the first identified patient showed the maternal origin of the mutation. In fact, we analyzed 56 somatic cell hybrid clones, using chromosome 8q11 markers and selected three clones containing only the duplicated chromosome 8 and three containing only the normal chromosome 8. Typing with a panel of polymorphic markers demonstrated that the duplication was of maternal origin and intrachromatidic (Supp. Table S1). Amplification and sequencing of exon 2 of the *SOX17* gene from the proposita, her parents, and selected hybrid clones demonstrated that in both the proposita and her mother, chromosome 8 carried the p.Y259N mutation, while the paternal chromosome 8 had a normal *SOX17* allele.

The screening for *SOX17* mutations was extended to 178 individuals with sporadic VUR and in 305 controls consisting of 135 children with nephrotic syndrome, 88 normal subjects without a history of urinary infection who were not further characterized, and 82 cord blood samples (610 chromosomes). Children with nephrotic syndrome were chosen because all had been followed for years with frequent urine analysis and echosonograpic evaluation. The same p.Y259N mutation was found in two patients of the sporadic VUR cohort, whereas two additional children carried different *SOX17* mutations (Table 1) (chi-squuare, *P*<0.01): one was a point mutation (p.G178C) producing a glycine to cysteine change at position 178, and the other (p.17Q_18SinsTQ) was characterized by an in-frame insertion of threonine–glutamine at position 17 (Fig. 1B and Table 1). Finally, p.Y259N was found in one normal blood donor DNA who was recruited from our Transfusion Unit without having any further clinical studies, including renal sonography (Table 1).

Two (p.G178C and p.Y259N) of the three mutations described here are localized between the HMG-box and the glycine–proline-rich segment at the C-terminal of the protein; the other is at the N-terminus near the HMG-box (Fig. 1B). Comparison of the predicted amino acid sequences indicates that human and mouse Sox17 share about 85% identity over their entire sequence, whereas zebrafish (Dr) Sox17 shows nearly perfect conservation only in the HMG box and the N- and C-terminal portions (Supp. Fig. S1). The Glycine 178 residue is conserved across all three species, while Tyrosine 259 is conserved between human and mouse (and the frog *Xenopus laevis*, data not shown); the 17Q_18S residues are located in the conserved N-terminal portion of the protein, but are not themselves conserved (Supp. Fig. S1).

### Analysis of SOX17 p.Y259N Protein Activity

**Figure 3:**
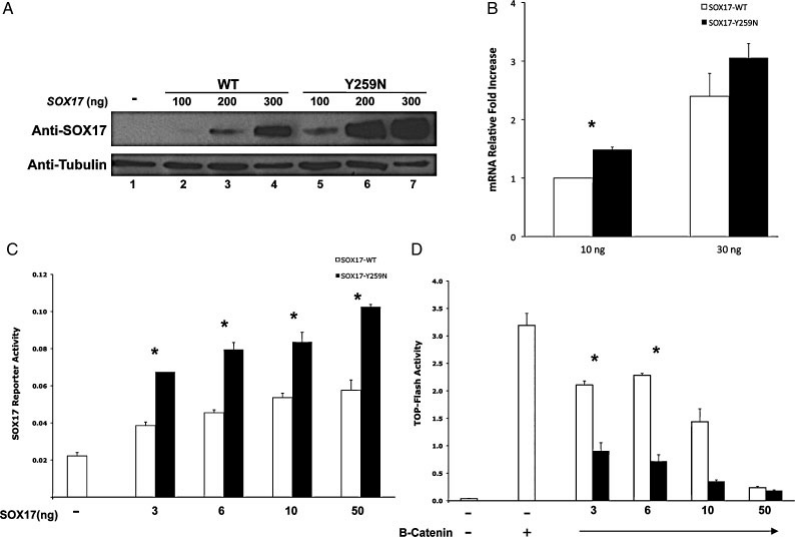
Impact of the p.Y259N mutation on *SOX17* mRNA and protein levels. **A:** Steady-state levels of *SOX17* protein are profoundly affected by the p.Y259N mutation. A total of 100, 200, or 300 ng of WT *SOX17* or p.Y259N *SOX17* expression plasmids were transfected into HEK293T cells and protein lysates were harvested after 48 hr and analyzed by Western blot with an anti-*SOX17* antibody, as well as an antitubulin antibody to assess protein loading. Comparison of SOX17-WT and SOX17-p.Y259N protein levels at equal dosage of transfecting DNA (lanes 2 and 5, 3 and 6, 4 and 7) suggests that the mutation increases SOX17 protein stability. Experiment was repeated four times and a representative blot is shown. **B:** *SOX17* mRNA levels measured by quantitative RT-PCR. *SOX17* mRNA levels in mock-transfected cells were 0.003 times that measured in cells transfected with 10 ng WT *SOX17* (data not shown). *N*=3; ^*^P=0.001; Error bars represent SEM. **C:** The effect of the p.Y259N mutation on the transcriptional activity of a *SOX17* reporter plasmid. *SOX17* expression plasmids were cotransfected with a *SOX17*-luciferase reporter plasmid. The SOX17-p.Y259N protein showed enhanced activity of the reporter at every transfection dosage, relative to the SOX17-WT protein. Data are shown as ratio of activities of firefly luciferase to control renilla luciferase. *N=*3; ^*^P<0.05; Error bars represent SEM. **D:** The effect of the p.Y259N mutation on the ability of SOX17 to repress Wnt signaling. *SOX17* expression plasmids were cotransfected with a TCF-luciferase reporter plasmid (TOP-flash) and an expression plasmid encoding a stabilized form of β-catenin. Repression of Wnt signaling was increased at every dosage with the mutant SOX17 protein, compared to the WT protein. Data are shown as ratio of activities of firefly luciferase to control renilla luciferase. *N*=3; ^*^P<0.05; Error bars represent SEM.

We investigated the impact of the p.Y259N mutation on SOX17 protein levels, SOX17 mRNA levels, transcriptional activity, and Wnt-inhibitory activity in the kidney cell line HEK293T. Western blot analysis of total cell extracts demonstrated that, at equal amounts of transfected DNA, the mutant SOX17-p.Y259N protein accumulated to much higher levels than SOX17-WT protein (Fig. 3A). In numerous experiments we observed between 6- and 20-fold increase in protein levels as measured by Western blot and densitometry analysis. These differences were not due differences in mRNA levels from the transfected plasmids, because quantitative RT-PCR on total RNA isolated from transfected cells revealed that mRNA levels of wild-type and *SOX17–*p.Y259N-transfected cells were largely comparable (Fig. 3B). In the example shown, there was a slight increase (1.4-fold) in levels of *SOX17–*p.Y259N mRNA relative to wild type, but this is minimal compared to the 6- to 20-fold increase in protein levels of SOX17–p.Y259N. We analyzed SOX17 transcriptional and Wnt-β-catenin repressing activity by cotransfection of *SOX17* with a *SOX17* reporter plasmid [Sinner et al., 2007] (Fig. 3C) and a Wnt reporter plasmid (Fig. 3D). In all cases, the p.Y259N mutant protein levels were significantly higher and this correlated with higher transcriptional activity as measured by the *SOX17* reporter plasmid. Moreover, SOX17–p.Y259N suppressed Wnt-β-catenin signaling activity two- to threefold better than wild-type SOX17 (Fig. 3D). However, when luciferase activity was approximately normalized to SOX17 protein levels, the p.Y259N mutation did not appear to alter the inherent transcriptional or Wnt/β-catenin inhibitory activity of SOX17 [Sinner et al., 2007; Wodarz and Nusse, 1998; Zorn et al., 1999]. Because we have previously demonstrated that SOX17 can interact with β-catenin and TCF/LEF proteins and target them for degradation, one likely interpretation for these data is that elevated p.Y259N mutant protein inappropriately suppresses the Wnt signaling pathway during kidney and urinary tract development causing the above-mentioned congenital defects.

## Discussion

A complex mechanism drives the development of the urinary tract in mammals. Both the kidney and urinary tract segments derive from the interaction of two tissue compartments of the embryonic mesoderm, that is, the ureteric bud (UB), derived from the Wolffian duct, and the metanephric mesenchyme (MM), that induces the metanephric mesenchyme to trans-differentiate into nephron epithelia [Vainio et al., 2002; Woolf et al., 2004]. These events are mediated by several soluble factors that act in a cooperative fashion either as pro- or antitubulogenic factors. Among the growing list of such molecules are the members of the fibroblast growth factor (FGF), transforming growth factor (TGF-β), and Wnt families, as well as GDNF, HGF, and EGF [Karihaloo et al., 2005]. β-Catenin is a key component of the canonical Wnt signaling pathway. Several Wnts are expressed during kidney development, and activation of the Wnt pathway can result in localization of β-catenin to the nucleus where it activates a pattern of gene expression that is required for normal nephron development [Karihaloo et al., 2005]. In fact, inhibiting β-catenin/Wnt signaling in the developing Wolffian duct results in mice with ectopic ureters and renal malformations [Marose et al., 2008].

We report here the identification of a novel heterozygous amino acid substitution in *SOX17* in two families and several other subjects with urinary tract malformations. Our results show that the p.Y259N mutation causes an increase in SOX17 protein levels in cultured kidney fibroblast cells. Tyrosine-259 is one of four putative tyrosine-phorphorylation sites, as predicted by analysis of SOX17 primary sequence [Blom et al., 1999]. The interactions between protein phosphorylation and the ubiquitin–proteasome system (UPS) are well documented [Gao and Karin, 2005; Hunter, 2007]. Specifically, a single phosphorylated residue can either target a protein for degradation or prevent its degradation by the UPS, depending on the protein being studied. We therefore speculate that the intracellular degradation of SOX17 is at least partially dependent on tyrosine-phosphorylation at residue 259.

*SOX17* is known to directly interact with transcriptional effectors of the Wnt signaling pathway, β-catenin, and TCF/LEF. *SOX17* physically interacts with β-catenin to regulate *SOX17* target genes [Sinner et al., 2004]. In addition, *SOX17* is a potent Wnt-signaling antagonist, and does so through direct physical interaction with both β-catenin and TCF/LEF factors [Sinner et al., 2007]. Formation of this trimeric complex leads to the degradation of both β-catenin and Tcf/Lef proteins through a GSK3-independent mechanism and loss of Wnt-signaling activity. Consistent with SOX17 acting to inhibit the Wnt/β-catenin pathway, our data showed that elevated *SOX17* p.Y259N protein inappropriately suppressed the Wnt signaling pathway in renal cells. Because mice lacking β-catenin in the developing kidney have symptoms of CAKUT, it is possible that altered levels of Wnt signaling during development could cause the observed congenital defects in these patients with CAKUT. Alternatively, elevated levels of Sox17 could effect urinary tract development through regulation of Sox17 target genes, of which several have been identified [Patterson et al., 2008; Sinner et al., 2004].

SOX17 is expressed throughout different stages of kidney development (http://www.gudmap.org) [Brunskill et al., 2008]. For example, in situ hybridization and microarray analysis shows that *Sox17* is highly expressed in bladder stroma and to a minor degree in the ureteric bud and in metanephric mesenchyme at E11.5 days after fertilization, a stage where *Wnt9b* acts to regulate ureteric branching [Carroll et al., 2005]. Other Wnt ligands, specifically Wnt2b, Wnt6, Wnt7b, and Wnt9b, as well as *Sox17,* are also expressed at later stages of kidney development such as in the S-shaped bodies, mesonephros, and metanephros, and later in the medullary collecting duct, renal medullary interstitium, maturing renal corpuscle, early proximal tubule, and cortical collecting duct.

Our in vitro analysis of SOX17 activity showed a clear correlation between SOX17 protein level and the degree of β-catenin repression, demonstrating a dose-dependent inhibitory effect of SOX17 on Wnt signaling. This is interesting because the proband's duplicated chromosome 8 contained two copies of the *SOX17–*p.Y259N mutation and would be predicted to exhibit a genetic “dose-dependent” increase in phenotypic severity. In fact, the patient with the duplication and two copies of *SOX 17–*p.Y259N, which would be predicted to have higher accumulation of SOX17 and more suppression of Wnt/β-catenin, had more severe developmental defects than the patients with one copy of *SOX 17–*p.Y259N.

To our knowledge, no mutation or disease has been associated with the *SOX17* gene in humans. Only two HMG box mutations were found in cell lines from colorectal cancers [Suraweera et al., 2006]. The presence of *SOX17* mutations in several patients with urinary tract anomalies suggests that *SOX17* is involved in regulatory and signaling pathways controlling the normal development of the urinary apparatus. The additional pathological features found exclusively in the proband of Family 1, such as psychomotor and language development delay and abnormal corpus callosum, were very likely due to the presence of the 13-Mb duplication spanning a portion of chromosome 8 containing over 30 genes. The reason for the rare association of *SOX17* functional mutations with diseases in humans probably resides on the fact that to date, over 20 SOX genes have been identified in vertebrates with closely related family members. For example, *SOX17*, *SOX7*, and *SOX18*, share high structural and functional similarities and have functional redundancies during development [Bowles et al., 2000; Sakamoto et al., 2007; Travers, 2000]. This indicates that Sox family members that are coexpressed in the same cell type will functionally substitute for one another.

In conclusion, the identification of *SOX17* mutations in individuals with duplication of the urinary tract and VUR has broad implications for the genetic workup in patients with kidney and urinary tract malformations. *SOX17* is the latest in a series of genes (*PAX2*, *ROBO2*) whose alteration induces partial defects of the VUR. Our observations also indirectly support a key role for *SOX17*, possibly via Wnt/β-catenin/Tcf signaling pathway, in kidney organogenesis, ureteric branching, and urinary tract development in humans [Zhang, 2003].
