# Supplementary material for: Mutations in SOX17 are Associated with Congenital Anomalies of the Kidney and the Urinary Tract
Source: Hum Mutat. 2010 Oct 19;31(12):1352–9. doi: 10.1002/humu.21378 (PMC3056145; doi:10.1002/humu.21378)
Supplement: Supplementary file 1 [file humu0031-1352-SD1.pdf]

Alignment:

```
                         .    :    .    :    .    :    .    :    .    :    .    :
Dr-Sox17       MSSPDAGYSSDDPSQTSSCSSVMMPGMGQCPWVDPLSPLSDSKSKHEKCSAAGP-----G    55
Hs-SOX17       MSSPDAGYASDDQSQTQSALPAVMAGLGPCPWAESLSPIGDMKVKGEAPANSGAPAGAAG    60
Mm-Sox17       MSSPDAGYASDDQSQPRSAQPAVMAGLGPCPWAESLSPLGDVKVKGEVVASSGAPAGTSG    60

                         .    :    .    :    .    :    .    :    .    :    .    :
Dr-Sox17       RGKSEPRIRRPMNAFMVWAKDERKRLAQQNPDLHNAELSKMLGKSWKALPMVDKRPFVEE   115
Hs-SOX17       RAKGESRIRRPMNAFMVWAKDERKRLAQQNPDLHNAELSKMLGKSWKALTLAEKRPFVEE   120
Mm-Sox17       RAKAESRIRRPMNAFMVWAKDERKRLAQQNPDLHNAELSKMLGKSWKALTLAEKRPFVEE   120

                         .    :    .    :    .    :    .    :    .    :    .    :
Dr-Sox17       AERLRVKHMQDHPNYKYRPRRRKQVKRNKRLEPSFPLPGMCDAKM-TLCTEGMSAGYSGA   174
Hs-SOX17       AERLRVQHMQDHPNYKYRPRRRKQVKRLKRVEGGF-LHGLAEPQAAALGPEGGRVAMDGL   179
Mm-Sox17       AERLRVQHMQDHPNYKYRPRRRKQVKRMKRVEGGF-LHALVEPQAGALGPEGGRVAMDGL   179

                         .    :    .    :    .    :    .    :    .    :    .    :
Dr-Sox17       GLPQYCENHTLFESYSLPTPDPSPMDAGTTEFFAQLQDQSAFSYHHQQEHHFQEQTNILN   234
Hs-SOX17       GLQFPEQGFPAGPPLLPPHMGGHYRDCQSLGAPPLDGYPLPTPDTSPLDGVDPDPAFFAA   239
Mm-Sox17       GLPFPEPGYPAGPPLMSPHMGPHYRDCQGLGAPALDGYPLPTPDTSPLDGVEQDPAFFAA   239

                         .    :    .    :    .    :    .    :    .    :    .    :
Dr-Sox17       DTHCHGNTQTLKSRQSHSIAYSNINTNTNSNLHAPINAQLSSINLQQVFHENANPQISHH   294
Hs-SOX17       PMPGDCPAAGTYSYAQVSDYAGPPEPPAGPMHPRLGPEPAGPSIPGLLAPPSALHVYYGA   299
Mm-Sox17       PLPGDCPAAGTYTYAPVSDYAVSVEPPAGPM--RVGPDPSGPAMPGILAPPSALHLYYGA   297

                         .    :    .    :    .    :    .    :    .    :    .    :
Dr-Sox17       PGTHLNIFNRSPSSSSHHAMTPA---YLNCPSTLDTFYNSSSQMKELSHCVSSHTHKQQS   351
Hs-SOX17       MGSPGAGGGRGFQMQPQHQHQHQ------HQHHPP-GPGQPSPPPEALPCRDGTDPSQPA   352
Mm-Sox17       MGSPAASAGRGFHAQPQQPLQPQAPPPPPQQQHPAHGPGQPSPPPEALPCRDGTESNQPT   357

                         .    :    .    :    .    :    .    :    .    :    .    :
Dr-Sox17       IAEAQSQASTATHSSGQMVDEVEFEHCLSFGVPSAPLPGSDLISTVLSDASSAVYYCGYN   411
Hs-SOX17       ELLGEVDRTEFEQYLHFVCKPEMGLPYQGHDSGVNLPDSHGAISSVVSDASSAVYYCNYP   412
Mm-Sox17       ELLGEVDRTEFEQYLPFVYKPEMGLPYQGHDCGVNLSDSHGAISSVVSDASSAVYYCNYP   417

                         .    :    .    :    .    :    .    :    .    :    .    :
Dr-Sox17       NS                                                            413
Hs-SOX17       DV                                                            414
Mm-Sox17       DI                                                            419
```

Supp. Figure S1. Alignment of vertebrate Sox17 protein sequences. Residues identical in all species are highlighted in yellow, those conserved only in two species are highlighted in blue. Tyrosine residues (Y) predicted to undergo phosphorylation are highlighted in red. The HMG box is indicated by a black line, the G/P-rich region by a red line. Red arrows indicated the location of the mutations. Tha Accession numbers for the sequences are as follows: Dr-Sox17 (NP_571362), Hs-SOX17 (NP_071899), Mm Sox17 (NP_035571). Dr: *Danio rerio*, Hs: *Homo sapiens*, Mm: *Mus musculus*.

Supp. Table S1. Molecular characterization of the duplicated chromosome 8q region in Family 1

Marker	Position (Kb)	Proband	Mother	Father	der(8)	chr. 8	Results
D8S1750	35470	216/220	216/218	216/220	ND	ND	N
D8S1821	38369	146/164	140/164	146	ND	ND	N
D8S268	41264	255/257	257	255/257	ND	ND	N
D8S1115	42544	164	161/164	161/164	ND	ND	U
Centromere							
D8S531	49074	117*/119	115/117	115/119	117	119	dup(M)
D8S601	53846	219/227*	223/227	219/231	227	219	dup(M)
D8S1737	54949	191	191	191	191	191	U
SOX17 E2 T/A	55534	T/A*	T/A	T	A	T	dup(M)
D8S509	55756	269*/271	269	271	269	271	dup(M)
D8S1828	56962	205	205	205	205	205	U
D8S260	60000	208/210	210	208	210	208	N

The proband, her mother and father, and two somatic cell hybrid clones containing either the duplicated chromosome 8 (der(8) or the normal chromosome 8 (chr. 8), were assayed. Duplicated alleles are indicated by an asterisk (*). N, normal chromosomal asset; U, uninformative; dup(M), duplication of maternal origin; ND, not done.
